# Impact of digital antenatal care intervention on paper-based antenatal care recordkeeping: a before-and-after study in primary healthcare facilities in Nepal

**DOI:** 10.1136/bmjopen-2024-086255

**Published:** 2025-03-15

**Authors:** Seema Das, Emma Radovich, Sulata Karki, Clara Calvert, Rajani Shakya, Loveday Penn-Kekana, Abha Shrestha, Biraj Man Karmacharya, Ona L McCarthy, Abha Shrestha, Oona M R Campbell

**Affiliations:** 1 Research and Development Division, Department of Public Health and Community Programs, Dhulikhel Hospital Kathmandu University Hospital, Dhulikhel, Nepal; 2 Emory University, Atlanta, Georgia, USA; 3 London School of Hygiene & Tropical Medicine, Faculty of Epidemiology and Population Health, London, UK; 4 The University of Edinburgh Usher Institute of Population Health Sciences and Informatics, Edinburgh, UK; 5 Department of Obstetrics and Gynecology, Kathmandu University School of Medical Sciences, Kathmandu, Nepal; 6 Department of Public Health and Community Programs, Kathmandu University School of Medical Sciences, Dhulikhel, Nepal; 7 Department of Community Medicine, Kathmandu University School of Medical Sciences, Dhulikhel, Nepal

**Keywords:** Quality Improvement, Clinical Decision-Making, Health Services

## Abstract

**Abstract:**

**Objective:**

To assess the impact of introducing electronic decision support systems (EDSS)—electronic data entry implemented alongside existing paper-based antenatal care (ANC) records—on the completeness and agreement of ANC records.

**Design:**

Two-phase cross-sectional (before and after) substudy of the mobile health integrated model of hypertension, diabetes and ANC (mIRA project) process evaluation.

**Setting:**

Four rural districts in Bagmati Province, Nepal, in 19 primary healthcare facilities.

**Participants:**

ANC records from pregnant women attending facilities before (n=136) and after (n=138) EDSS implementation.

**Main outcome measures:**

For selected indicators in the ANC card and ANC register, we estimated the percentage completeness (any value recorded) and agreement (whether values matched) before and after EDSS implementation. We also reported the completeness of indicators in the EDSS and calculated the agreement between the ANC card and EDSS. χ^2^ or Fisher’s exact test, as appropriate, was used to assess differences in completeness before and after implementation.

**Results:**

Completeness of paper-based ANC records was high before implementation (>90%) for all indicators, except tetanus vaccination (<80%). After EDSS implementation, there was >15% improvement in the completeness of tetanus vaccination date in paper-based ANC records (77.0%–96.4% for ANC cards and 81.9%–98.9% for ANC register). Agreement between the ANC card and ANC register increased slightly for all indicators after implementation, and the tetanus vaccination date showed the largest increase (38.2%–57.2%). Indicator completeness in the EDSS was low, ranging from 38.2% to 88.7%.

**Conclusion:**

We found slight improvements in the completeness and agreement of paper-based ANC records following EDSS implementation. The lower percentage of completeness in the EDSS suggests that any large-scale implementation should consider how to integrate digital and paper-based records to decrease the data entry burden on ANC providers. However, the study’s small sample size limited the ability to examine variation in effects.

**STRENGTHS AND LIMITATIONS OF THIS STUDY**

- The findings of this study will be of interest to evaluators of digital interventions more broadly about the potential unintended consequences of interventions.
- It offers both a methodological contribution (how to examine potential negative impacts of digital intervention) and an empirical contribution (adverse effects on documentation not seen, but highlights the complexity of attempting to introduce a parallel record-keeping system).
- Our study was unable to examine accuracy due to logistic constraints in using observations of care as a ‘gold standard’ for comparison.
- This study was limited to only primary healthcare facilities in Province 3 of Nepal, and the sample size was small for stratified analysis by EDSS.
- Despite the small sample size for stratified analysis by electronic decision support systems (EDSS), we found meaningful and statistically significant differences in indicator completeness between the paper-based records and each EDSS.

## Introduction

 The reduction of maternal mortality remains a high priority, with Sustainable Development Goal 3 setting a target to reduce the global maternal mortality ratio to <70/100 000 live births by 2030.^1^ According to the 2021 National Population and Housing Census, the maternal mortality rate of women in Nepal was 151/100 000 live births.^2^ Antenatal care (ANC) plays a vital role in the reduction of maternal and perinatal mortality and morbidity.^3^ ANC is aimed at ensuring all pregnant women receive screening tests, have early detection and prevention of complications and adequate management of pre-existing maternal diseases and monitoring throughout the pregnancy.^4 5^ However, in some settings such as Nepal, there is high coverage of ANC services, but a gap remains in the quality of care provided.^6^ ANC quality is often measured by the number of visits, early initiations of care and receiving recommended interventions.^5^ Observations of ANC consultations in healthcare facilities in Nepal found that recommended care components were performed less often in lower-level health facilities.^7^ For example, 52.5% of first ANC consultations at primary healthcare centres included urine testing, compared with 33.5% in health posts.^7^

Accurate and complete recordkeeping is important in ANC for providers to know what care components are needed and to support continuity of care over different ANC visits during pregnancy.^8^ Medical recordkeeping is critical to assuring quality of care.^9^ Records aid in assessing and managing an individual patient’s care and also contribute to monitoring and improving service delivery.^9 10^ In Nepal, pregnant women’s handheld ANC card and facility ANC register are the basic sources of information for mother and child health conditions. Both ANC card and ANC register record women’s health information and details of ANC visits (as well as delivery and postnatal care). Pregnant women keep their ANC card with them and are asked to bring it to every ANC visit to be filled in by the ANC provider. The ANC card allows women to understand pregnancy progress and their next appointment date.^11^ The ANC register is the facility-based register where ANC providers record every ANC visit; it includes fewer information fields than the ANC card. These two documents aid in the systematic recording of information during each ANC consultation.

This study is part of the process evaluation of the mobile health integrated model of hypertension, diabetes and ANC (mIRA) project. The mIRA project implemented and compared two electronic decision support systems (EDSS) which aimed to improve the quality of ANC in primary healthcare facilities in Nepal.^12^ The first EDSS, mIRA EDSS, was designed by the Public Health Foundation of India, Dhulikhel Hospital, Kathmandu University Hospital, and the London School of Hygiene & Tropical Medicine.^12^ The second EDSS, the WHO EDSS, was developed by WHO to facilitate the adoption of the WHO ANC guidelines and was subsequently customised to Nepal.^13^ Both EDSS provided checklists and prompts, based on national protocols, to improve adherence to routine ANC guidelines and facilitate the detection of pregnancy complications.^14^ ANC providers were expected to use the EDSS during ANC consultations with pregnant women, recording clinical examinations and test results in the EDSS. Barriers such as increased workload, fragmentation of workflow, lack of technical competency and poor acceptance of EDSS by care providers often hinder the implementation and effects of EDSS.^15 16^ The results of the mIRA project evaluation are reported elsewhere,^14^ but the EDSS largely did not result in the desired quality improvements. However, the evaluation was also interested in examining the additional effects of the intervention on recordkeeping.^12^

The two EDSS and associated electronic data entry were implemented alongside existing paper-based ANC records during the research project, adding additional record-keeping requirements for ANC providers as a part of the intervention. With the addition of electronic recordkeeping, one potential unintended consequence could be a change in completeness and/or agreement of existing paper-based ANC records.

While other studies have examined either the completeness and accuracy of maternal health services data in district health information management systems,^17 18^ or agreement between self-reported questionnaires and ANC cards.^19^ To our knowledge, no studies to date have specifically evaluated the impact of introducing an EDSS on existing documents, highlighting a critical gap in research exploring the impacts of additional electronic recordkeeping on the completeness and agreement of existing paper-based records. Thus, the objective of this study was to assess change in the completeness and agreement of the ANC card and ANC register before and after EDSS implementation. The study also aimed to examine the completeness of the data in the EDSS and the agreement between the ANC card and the EDSS.

## Methods

### Study setting and design

The study was conducted in four rural districts in Bagmati Province, Nepal in 19 primary healthcare facilities, participating in the mIRA project. Facilities included government Health Posts, government Primary Health Care Centers and Dhulikhel Hospital Outreach Centers—which are non-governmental clinics similar in capacity and structure to Primary Health Care Centers. The 19 health facilities were paired by facility type and randomly allocated to receive a tablet with the EDSS software: 10 with the mIRA EDSS and the remaining 9 with the WHO EDSS.^14^

This was a two-phase cross-sectional (before and after) substudy of the mIRA process evaluation^12^ with data collection before implementation conducted from December 2021 to March 2022 and data collection after implementation conducted from June to August 2022 (about 3–5 months after EDSS implementation).

### Intervention

mIRA EDSS and WHO EDSS aimed to improve providers’ adherence to routine ANC guidelines and detect and manage high-risk pregnancies.^12 14^ The WHO EDSS focused on screening and referral, while the mIRA EDSS provided bespoke diagnosis and treatment prompts for gestational diabetes, hypertension in pregnancy and anaemia.^14^ All participating facilities received a tablet with the allocated EDSS software installed, a SIM card for mobile data and on-site technical support for the first month of implementation.^14^ One ANC provider from each participating facility—mainly auxiliary midwives and staff nurses—was selected by the local municipality to attend a 3 day training workshop on how to use their allocated EDSS, but all ANC providers at the participating facilities involved in conducting ANC consultations and managing ANC records were eligible to use the EDSS. About 62 ANC providers participated, 19 of whom received in-person training. The working experience of ANC providers ranged from 1 to 25 years, with the majority having more than 2 years of work experience.

More details about the intervention, including its mechanism and implementation, are available elsewhere.^12 14 20 21^

### Participants

The study included data from the ANC records of pregnant women. All pregnant women aged 18 years and above who had attended a participating health facility for a regular ANC consultation during the data collection period were approached (until the required sample size was met) for consent and inclusion in the study. Pregnant women attending facilities only to get a blood test or ultrasound services were excluded from the study.

### Sample size

A sample size of 138 pregnant women’s records in each round of data collection (before and after EDSS implementation) was estimated to provide adequate power (80%) at the significance level of 0.05 to detect the difference of ≥15% in completeness before and after EDSS implementation, with an assumption of 65% initial prevalence of completeness. This initial prevalence of completeness was based on an analysis of data from handheld ANC cards that were extracted in the pilot phase of the mIRA study; two key fields (date of last menstrual period and estimated delivery date) were completed in 60%–65% of the ANC cards. The sample size was potentially underpowered for stratifying by EDSS.

### Definitions and data collection tool

We conducted a rapid literature review and referred to the United Kingdom’s National Health Service guidelines^22^ to identify record-keeping dimensions and approaches to assess the impacts of EDSS implementation on the quality of paper-based ANC records and to develop our data collection tool.^9 12 15^ Another dimension of data quality—accuracy—could not be assessed in our study due to logistical constraints in comparing the paper-based records to a gold standard of observations of the care provided.

We selected indicators that were common to both the ANC card and ANC register as well as some additional indicators common in the EDSS and ANC card. Nine indicators on the ANC card and seven indicators from the ANC register were selected. The following indicators were available in both the ANC card and ANC register: (1) date of ANC register, (2) ANC registration number, (3) woman’s age, (4) last menstrual period date, (5) parity, (6) whether the first dose of tetanus vaccination was received and, if so, (7) tetanus vaccination date. The pregnant woman’s weight at their first ANC visit (in kilograms) and blood pressure measurement (systolic blood pressure/diastolic blood pressure) for that day’s visit were additionally available in the ANC card. All nine selected indicators from the ANC card were also available in mIRA EDSS; however, three indicators—the date of ANC registration, the pregnant women’s weight at their first visit and tetanus vaccination date—were not present in WHO EDSS (see online supplemental additional file 1).

The outcomes of completeness and agreement were measured across the three data sources: ANC card, ANC register and EDSS record. Completeness referred to whether any value for the selected indicators was recorded in the ANC card and ANC register, and in the EDSS record (second round of data collection only). Completeness was a binary indicator, with any value recorded for the selected indicators coded as ‘yes’ or ‘no’. The agreement referred to whether the value recorded for each selected indicator exactly matched what was recorded in the ANC card and ANC register, before and after the implementation. Additionally, an agreement between the ANC card and the EDSS indicators was compared separately.

### Data collection

The first round of data collection was carried out between December 2021 and March 2022, and the second round of data collection was carried out between June and August 2022 after approximately 3–5 months of EDSS implementation. In each facility, a research assistant was stationed for up to 7 days and consented to eligible pregnant women attending ANC visits. The research assistant took a photo of the pregnant woman’s ANC card or, time permitting, directly extracted the information into the paper-based data collection tool, while the woman was still at the facility and then extracted the same woman’s information from the facility’s ANC register at the end of the same day. In the second round, the research assistant additionally extracted data from the EDSS tablets on the day of in-person data collection in a facility. For women missing an EDSS entry, the first author (SD) checked the data that were stored in software (backend data) for the same woman on the same date of the ANC visit after 1 week of data collection, to account for later data entry in the EDSS after the woman’s ANC visit. To ensure that data of the same women were obtained, women’s name, phone number, place, date of visit and husband’s name were linked across the three data sources. Following each round of data collection, data from the paper-based data collection tool was entered into Kobo Toolbox (www.kobotoolbox.org) by the research assistants. Direct data extraction into Kobo Toolbox was not possible because of the lack of tablets for data collection.

### Data quality and management

We piloted and modified the data collection tool and trained the research assistants before data collection. Regular check-ins of research assistants were done to monitor any problems with data collection and to ensure the quality of data. During the second round of data collection, about 10% of the paper-based data collection forms were checked against the photos of the ANC card and ANC register. Unfortunately, cross-verification of data extraction was not done in the first round of data collection because of the unavailability of photos of the ANC card and ANC register. However, cross-verification in the second round of data collection showed <5% error in extracted data when compared with photos of the ANC card and ANC register. After entering the data in the Kobo Toolbox, Kobo entries data were again cross-checked with paper-based data collection forms; minimal errors were corrected.

### Data analysis

The data obtained were cleaned and coded to facilitate data analysis. The statistical analysis was performed using the Statistical Package for Social Science V. 24. For completeness, we calculated the number and percentage of records with any value recorded for the selected indicators in the ANC card and ANC register before and after implementation. We used χ^2^ tests to assess the evidence for differences in the completeness of the ANC card and ANC register before and after EDSS implementation for date of registration, tetanus vaccination first dose and first ANC visit weight. For the remaining indicators, we instead used Fisher’s exact test to look at the evidence for changes in completeness due to small numbers. Similarly, the differences in the completeness of indicators for the paper-based ANC records and each EDSS were also computed using Fisher’s exact test due to the small numbers.

For agreement, we calculated the percentage of women where the value matched for selected indicators from the ANC cards and the ANC register. We also calculated the agreement between the ANC card and what was recorded in the WHO EDSS and mIRA EDSS. The ANC card was selected for the agreement standard based on formative research where ANC providers primarily depend on information provided on the ANC card to guide their actions. A study conducted in Brazil was additionally used as a reference.^19^ Agreement was only calculated for women where there was a value entered in both data sources. For two of the indicators—any tetanus vaccination received and most recent blood pressure—the agreement was not calculated because both were ‘yes’ or ‘no’ responses and were recorded differently in the EDSS compared with the ANC card, and therefore values could not be matched.

### Patient and public involvement

No patient or public was directly involved in the design and conduct of this study. However, patients and providers were involved in formative research for the mIRA project evaluation.^14^ Findings from this study were shared with healthcare providers and stakeholders as part of the mIRA project.

## Results

A total of 136 records were collected in the first round and, in the second round, 138 records were collected (76 records from mIRA EDSS facilities and 62 records from WHO EDSS facilities). Before implementation, the majority of women’s records were of aged 20–25 years (46%), followed by 26–30 years (32.4%) and the lowest for >30 years (9.6%). The ANC register record showed a similar distribution: 20–25 years (44.1%), 26–30 years (30.1%), >30 years (8.8%). After EDSS implementation, a slight variation was observed in paper-based ANC records. The lowest age group was <20 years, accounting for approximately 10% of the records, while the highest age group was 20–25 years with approximately 45% of the records. After EDSS implementation, about 28% of the records represented first ANC visits, whereas most of the records (72%) were for follow-up visits.

### Completeness

Before EDSS implementation, the completeness of ANC cards was above 90% for all indicators, except for the first tetanus vaccination and tetanus vaccination date (table 1). The proportion of completeness varied from 73.5% (tetanus vaccination first dose) to 100% (women’s age). This was similar to the selected indicators of the ANC register where the percentage of completeness varied from 61.0% (first tetanus vaccination) to 96.3% (women’s age). As shown in table 1, there was either no difference in completeness or some evidence of improvement in completeness after EDSS implementation. There was more than a 15% improvement in the completeness of first tetanus vaccination date records after EDSS implementation in both paper-based ANC records (77.0%–96.4% (*p*<0.001) for ANC card and 81.9%–98.9% (*p*<0.001) for ANC register). For both paper-based ANC records, parity increased in completeness after implementation (92.6%–99.3% (*p*=0.005) for ANC card and 81.6%–92.0% (*p*=0.011) for ANC register).

**Table 1:** Completeness of selected indicators of ANC card and ANC register before (n=136) and after EDSS implementation (n=138)

Indicators	ANC card completenessn (%)				ANC register completenessn (%)			
	Before	After	% differences	P value	Before	After	% differences	P value
Date of ANC registration	127 (93.4)	130 (94.2)	+0.8	0.778	127 (93.4)	135 (97.8)	+4.4	0.072
ANC registration number	130 (95.6)	137 (99.3)	+3.7	0.065	126 (92.6)	132 (95.7)	+3.1	0.289
Woman’s age	136 (100.0)	137 (99.3)	−0.7	1.000	131 (96.3)	136 (98.6)	+2.3	0.280
Last menstrual period date	134 (98.5)	137 (99.3)	+0.8	0.621	126 (92.6)	135 (97.8)	+5.2	0.044
Parity	126 (92.6)	137 (99.3)	+6.7	0.005	111 (81.6)	127 (92.0)	+10.4	0.011
Tetanus vaccination received (first dose)	100 (73.5)	111 (80.4)	+6.9	0.174	83 (61.0)	91 (65.9)	+4.9	0.398
*Tetanus vaccination date*	77 (77.0)	107 (96.4)	+19.4	<0.001	68 (81.9)	90 (98.9)	+17.0	<0.001
First ANC visit weight (kg)	131 (96.3)	132 (95.7)	−0.6	0.777	Not included†	Not included†	NA	NA
Blood pressure measurement (current visit)	127 (93.4)	134 (97.1)	+3.7	0.167	Not included†	Not included†	NA	NA

* Tetanus vaccination date was calculated out of total Tetanus vaccination first dose received.

† First ANC visit weight and blood pressure measurement was not available in ANC register.

ANCantenatal careEDSSelectronic decision support systems

Table 2 shows the completeness of indicators after EDSS implementation, comparing the ANC card, ANC register and each EDSS, stratified by whether the records were from facilities implementing the WHO EDSS or from facilities implementing the mIRA EDSS. The indicators were more complete in the paper-based records compared with EDSS records for both the mIRA EDSS and the WHO EDSS, except for the first dose of tetanus vaccination which was more complete in WHO EDSS (88.7%, compared with 74.2% in ANC card and 58.1% in ANC register, *p*<0.001). Compared with the ANC card and ANC register, indicators in the mIRA EDSS were less complete, and completion was lowest for the first tetanus vaccination received (ANC card=85.5%, ANC register=73.7% and mIRA EDSS=38.2%, *p*<0.001).

**Table 2:** Completeness of selected ANC indicators after implementation of WHO EDSS (n=62) and mIRA EDSS (n=76)

Indicators	Records from WHO EDSS facilities				Records from mIRA EDSS facilities			
	ANC cardn (%)	ANC registern (%)	WHO-EDSSn (%)	P value	ANC cardn (%)	ANC registern (%)	mIRA-EDSSn (%)	P value
Date of ANC registration	56 (90.3)	62 (100.0)	Not included*	<0.001	74 (97.4)	73 (96.1)	66 (86.8)	0.102
ANC registration number	62 (100)	61 (98.4)	55 (88.7)	0.006	75 (98.7)	71 (93.4)	66 (86.8)	0.017
Woman’s age	62 (100.0)	62 (100.0)	55 (88.7)	<0.001	75 (98.7)	74 (97.4)	66 (86.8)	0.006
Last menstrual period date	62 (100.0)	62 (100.0)	55 (88.7)	<0.001	75 (98.7)	73 (96.1)	66 (86.8)	0.013
Parity	61 (98.4)	58 (93.5)	55 (88.7)	0.051	76 (100.0)	69 (90.8)	65 (85.5)	<0.001
Tetanus vaccination received (first dose)	46 (74.2)	36 (58.1)	55 (88.7)	<0.001	65 (85.5)	56 (73.7)	29 (38.2)	<0.001
[Table-fn T2_FN2]Tetanus vaccination date†	43 (93.5)	34 (94.4)	Not included*	0.050	64 (98.5)	56 (100.0)	29 (100.0)	1.000
First ANC visit weight (kg)	58 (93.5)	Not included‡	Not included*	NA	74 (97.4)	Not included‡	65 (87.8)	0.025
[Table-fn T2_FN4]Most recent blood pressure§	62 (100.0)	Not included‡	55 (88.7)	0.013	72 (94.7)	Not included‡	55 (72.4)	<0.001

* Date of ANC registration, tetanus vaccinate date, first ANC visit weight were not available in WHO EDSS.

† Tetanus vaccination date was calculated out of total tetanus vaccination first dose received.

‡ First ANC visit weight and most recent blood pressure measurement was not available in ANC register.

§ Women’s blood pressure on visit day of data collection.

ANCantenatal careEDSSelectronic decision support systems

### Agreement

Table 3 shows agreement between values recorded in the ANC card and the ANC register for each indicator, before and after EDSS implementation. The percentage agreement of the ANC registered with the ANC card ranged from 38.2% (tetanus vaccination date) to 91.2% (women’s age) before EDSS implementation, and slight improvement was observed across all indicators after EDSS implementation. Only women’s age showed >90% agreement before and after EDSS implementation. Tetanus vaccination date showed the largest percentage increase in agreement before and after implementation (38.2%–57.2%).

**Table 3:** Agreement of values of indicator of ANC register with ANC card before and after EDSS implementation

Indicators	Before EDSS implementation		After EDSS implementation	
	% value present in both ANC card and ANC register	% ANC register value matched with ANC card	% value present in both ANC card and ANC register	% ANC register value matched with ANC card
Date of ANC registration	87.5	69.9	92.0	78.3
ANC registration number	90.4	86.0	94.9	89.9
Woman’s age	96.3	91.2	97.8	92.0
Last menstrual period date	91.9	82.4	97.1	87.0
Parity	78.7	75.7	91.3	83.3
Tetanus vaccination received (first dose)	58.8	*NA	63.8	*NA
Tetanus vaccination date†	44.1	38.2	63.0	57.2

* Tetanus vaccination first dose given (yes/no).

† Tetanus vaccination date was calculated out of total tetanus vaccination first dose received.

ANCantenatal careEDSSelectronic decision support systems

Agreement between the WHO EDSS and the ANC card varied from 56.5% (parity) up to 82.3% (diastolic blood pressure) (table 4). Agreement between the mIRA EDSS and the ANC card ranged from 30.3% (tetanus vaccination date) up to 81.6% (parity) (table 4). Both systolic and diastolic blood pressure had 59.2% agreement in the mIRA EDSS compared with 77.4% and 82.3%, respectively, in the WHO EDSS.

**Table 4:** Agreement of values of the indicator of WHO EDSS and mIRA EDSS with ANC card after implementation

Indicators	WHO EDSS		mIRA EDSS	
	% value present in both ANC card and WHO EDSS	% WHO EDSS value matched with ANC card	% value present in both ANC card and mIRA EDSS	% mIRA EDSS value matched with ANC card
Date of ANC registration	Not included*	Not included*	84.2	64.5
ANC registration number	88.7	75.8	85.5	73.7
Woman’s age	88.7	67.7	85.5	80.3
Last menstrual period date	88.7	72.6	86.8	78.9
Parity	87.1	56.5	85.5	81.6
Tetanus vaccination received (first dose)	69.4	†NA	52.6	†NA
[Table-fn T4_FN3]Tetanus vaccination date‡	Not included*	Not included*	38.2	30.3
First ANC visit weight (kg)	Not included*	Not included*	82.9	69.7
[Table-fn T4_FN4]Most recent blood pressure§				
Systolic blood pressure	88.7	77.4	75.0	59.2
Diastolic blood pressure	88.7	82.3	75.0	59.2

* Date of ANC registration, tetanus vaccinate date, first ANC visit weight were not available in WHO EDSS.

† Recorded differently in the EDSS compared tocompared with the ANC card and therefore values could not be matched.

‡ Tetanus vaccination date was calculated out of total tetanus vaccination first dose received.

§ Women’s blood pressure on visit day of data collection.

ANCantenatal careEDSSelectronic decision support systems

## Discussion

To our knowledge, this is the first study evaluating the completeness and agreement between paper-based ANC records and electronic records in Nepal. We found indicators in the ANC card and ANC register showed high completeness before and after EDSS implementation. Before EDSS implementation, the percentage agreement was relatively high between the ANC card and ANC register for most indicators, with a few exceptions, including parity and tetanus vaccination received. The percentage agreement of indicators in the ANC register compared with the ANC card slightly increased after EDSS implementation. Completeness and agreement of indicators in both EDSS were low, suggesting that recordkeeping in the ANC card and ANC register was prioritised.

Unsurprisingly, we found high completeness of the paper-based records due to the central role they play in the healthcare system. The government of Nepal provides cash incentives to pregnant women on completion of required/protocol ANC visits and institutional birth, as documented on the ANC card.^23^ The ANC registers are mainly used by ANC providers to calculate numbers for monthly reporting (eg, the monthly number of first ANC contacts, the monthly number of pregnant women completing protocol ANC visits and a number of tetanus vaccine doses given).^24^

We found improvements in both completeness of and agreement between, the ANC card and ANC register following the introduction of the EDSS; however, we also found a lower percentage of completeness in the EDSS compared with the paper-based records and a lower percentage of agreement between the EDSS and the ANC card indicators. As the EDSS was designed to provide reminders and prompts to the ANC providers during ANC consultations,^14^ the EDSS may have prompted ANC providers to additionally fill in additional items in the paper ANC records explaining improvement in completeness after EDSS implementation.^15^ Also understood the intervention as about recordkeeping,^14^ which may have increased providers’ attention towards the paper-based records.^21^

There are several possible reasons for the comparatively low completeness of and agreement with ANC cards, and the data entered into the EDSS. First, the EDSS were implemented as part of a time-limited research project, and not a government initiative, potentially explaining why ANC providers did not prioritise filling these in. Other studies from the mIRA project found that government-initiated work was perceived as a legitimate part of their duties, and healthcare providers prioritised it.^14^ The EDSS were not linked with the government District Health Information System and hence did not help ANC providers in monthly reporting.^14^ A study conducted in India also recommended that the monthly reporting system should be added to electronic health records to avoid duplication of efforts.^25^ Second, in the mIRA EDSS, it was possible for the user to skip to the next section and avoid entering data for all fields, resulting in low completeness. Potentially, relatively low information technology literacy may have led to lower utilisation of EDSS and influenced completeness and agreement.^21^ A study conducted in hospitals in Ethiopia found that computer literacy was associated with better use of electronic records.^26^ In addition, the ease of use and functionality of the EDSS may have impacted its use.^27^ Enhancing the completeness and agreement of data in EDSS requires sufficient technical support and practice, and regular monitoring of data by government authorities.^25^

We did observe slightly different patterns for one of the indicators: tetanus vaccination. This indicator had low initial completeness in both the ANC card and ANC register (<80%), which was lower than expected, with results from the Demographic and Health Survey 2022 showing that 93% women aged 15–49 years who had recently given birth were immunised with tetanus vaccination.^28^ Although there was some variation by age, women aged 35–49 years had lower levels (82%).^28^ The low completeness in the paper-based ANC records in our study may have been because women missed ANC visits to receive the dose at the designated time.^29^ ANC providers also use outpatient cards and/or the ANC register for documentation of services received in early pregnancy, later copying data to the ANC card.^14 21^ This might lead to instances where the first dose tetanus vaccination provided in the first trimester was either missed or incorrectly copied to other records. However, there was some evidence of improvement in the recording of tetanus vaccination in paper-based records after EDSS implementation. For instance, in the facilities where the tetanus vaccination was provided on a designated day, the immunisation register was used to record the tetanus vaccination status, which could affect completeness and agreement in ANC records. If a pregnant woman forgot to bring her ANC card on the immunisation day, during their next ANC consultation, the tetanus vaccination section in the EDSS would have reminded the ANC providers to update tetanus vaccination status in the paper-based ANC records and in the EDSS.

Understanding the imperatives and priorities of healthcare providers is crucial for the long-term sustainability of EDSS interventions.^30^ In the mIRA project, ANC providers had to document the same information in two paper records along with additional electronic records, increasing the recording burden. Due to workload and time constraints, ANC providers might have missed or deprioritised recording in EDSS. The mIRA project evaluation found ANC providers did not use the EDSS consistently for all ANC visits and often copied information into the EDSS after women left the facility, relying on their memory or photos taken of the ANC card.^21^ A qualitative study exploring the implementation of electronic records in a maternity unit found that participants expressed frustration with the additional time required to manage both paper and electronic records.^31^ Likewise, a review article showed that one of the common concerns for healthcare providers regarding the use of digital healthcare technologies was increasing hours or workload.^32^ Further efforts to introduce electronic recordkeeping should replace or limit the additional burden of paper-based recordkeeping. Easing the burden of recordkeeping and ensuring its relevance would be helpful in motivating ANC providers to see documentation as an important task and part of assuring the quality of care.^9^ Electronic records can be designed to link with different health facilities, which could make it easier to transfer information from one facility to another, enhancing easy access to records and continuity of care.^25^ Furthermore, ensuring interoperability and user-friendly interfaces is essential for optimising the utilisation of electronic records.^30^

### Limitations

The results of this study contribute to future implementation of EDSS in low-resource settings, although some limitations can be found. Despite the small sample size and limited power for stratified analysis by EDSS, we found meaningful improvements in indicator completeness between the paper-based records for each EDSS. However, we were unable to fully account for variation within and between health facilities in the effects of introducing the two EDSS on paper-based recordkeeping. Other studies from the evaluation noted that ANC documentation was managed differently in the participating facilities, with, for example, some facilities sharing EDSS and other recordkeeping across multiple ANC providers and other facilities allocating documentation to only the provider who attended the EDSS training workshop.^21^ How recordkeeping is managed and shared within teams of ANC providers should be considered when designing and sampling future research studies on the effects of EDSS implementation on paper-based recordkeeping.

Our study was unable to examine accuracy due to logistic constraints to using observations of care as a ‘gold standard’ for comparison, and further research is important to assess the accuracy of information in different forms of recordkeeping. Similarly, we were unable to cross-verify data extraction of the paper-based records during the first round (before EDSS implementation) data collection, though we note there was a negligible amount of error in the second round (after EDSS implementation) data collection. Data entry from the paper-based tool to Kobo Toolbox for analysis may have introduced errors, though quality checks and management aimed to minimise this.

### Conclusion

The introduction of EDSS did not have a negative impact on paper-based ANC records. The additional recordkeeping required by the EDSS intervention resulted in general improvements in the completeness and agreement of ANC cards and ANC registers. The completeness of the selected indicators in the EDSS tended to be lower than that of the paper-based records. This study provides preliminary insights into the impact of EDSS on existing paper-based ANC records due to the small sample size and levels in the data. Large-scale EDSS implementation should also consider how to integrate electronic and paper-based records to decrease the documentation burden on healthcare providers, and the standard of records should be audited on a regular basis. Digital records are widely used in high-income countries to improve documentation accuracy, enhance patient care and provide better organisation and access to data. The increasing implementation of electronic health records in low-resource settings increases the demand for evidence to understand the feasibility and impacts of digital health interventions in real-world settings.

## supplementary material

10.1136/bmjopen-2024-086255 online supplemental file 1

## Data Availability

Data are available on reasonable request.
