# Supplementary material for: Impact of digital antenatal care intervention on paper-based antenatal care recordkeeping: a before-and-after study in primary healthcare facilities in Nepal
Source: BMJ Open. 2025 Mar 15;15(3):e086255. doi: 10.1136/bmjopen-2024-086255 (PMC11911671; doi:10.1136/bmjopen-2024-086255)
Supplement: online supplemental file 1 [file bmjopen-15-3-s001.pdf]

Data collection tool

Audit of Record Keeping – Nepal data extraction tool

Facility code:	
Pregnant woman identification number (PID):	
Date of audit:	
Fieldworker name:	

Variable		ANC Handheld Card		Facility ANC register		EDSS (Round 2 data collection only)	
		A. Does the field contain a value?	B. If yes, enter field value:	C. Does the field contain a value?	D. If yes, enter field value:	E. Does the field contain a value?	F. If yes, enter field value:
1	Date of ANC registration	☐Yes → 1B<br>☐No →2		☐Yes → 1D<br>☐No →2		☐Yes → 1F<br>☐No →2	
2	ANC registration number	☐Yes → 2B<br>☐No →3		☐Yes → 2D<br>☐No →3		☐Yes → 2F<br>☐No →3	
3	Woman's age	☐Yes → 3B<br>☐No →4		☐Yes → 3D<br>☐No →4		☐Yes → 3F<br>☐No →4	
4	Last menstrual period (LMP) date	☐Yes → 4B<br>☐No →5		☐Yes → 4D<br>☐No →5		☐Yes → 4F<br>☐No →5	
5	Parity	☐Yes → 5B<br>☐No →6		☐Yes → 5D<br>☐No →6		☐Yes → 5F<br>☐No →6	
6	TT/TD-1 vaccination received (first dose)	☐Yes → 7<br>☐No →8		☐Yes → 7<br>☐No		☐Yes → 7<br>☐No →8	
7	TT/TD-1 vaccination date	☐Yes → 7B<br>☐No →8		☐Yes → 7D<br>☐No		☐Yes → 7F<br>☐No →8	
8	ANC visit 1: weight in KG	☐Yes → 8B<br>☐No →9				☐Yes → 8F<br>☐No →9	
9	Most recent ANC visit: Blood pressure	☐Yes → 9B<br>☐No				☐Yes → 9F<br>☐No	
