# Supplementary material for: Impact of digital antenatal care intervention on paper-based antenatal care recordkeeping: a before-and-after study in primary healthcare facilities in Nepal
Source: BMJ Open. 2025 Mar 15;15(3):e086255. doi: 10.1136/bmjopen-2024-086255 (PMC11911671; doi:10.1136/bmjopen-2024-086255)
Supplement: Supplementary file 2 [file bmjopen-2024-086255.reviewer_comments.pdf]

PEER REVIEW HISTORY

BMJ Open publishes all reviews undertaken for accepted manuscripts. Reviewers are asked to complete a checklist review form and are provided with free text boxes to elaborate on their assessment. These free text comments are reproduced below.

ARTICLE DETAILS

Title (Provisional)

The impact of digital antenatal care intervention on paper-based antenatal care recordkeeping: a before-and-after study in primary healthcare facilities in Nepal

Authors

Das, Seema; Radovich, Emma; Karki, Sulata; Calvert, Clara; Shakya, Rajani; Penn-Kekana, Loveday; Shrestha, Abha; Karmacharya, Biraj Man; McCarthy, Ona L; Shrestha, Abha; Campbell, Oona

---

VERSION 1 - REVIEW

**Reviewer**	**1**
**Name**	**Anraad , Charlotte**
**Affiliation**	**Maastricht University, Work and Social Psychology**
**Date**	**23-May-2024**
**COI**	**None**

First, I would like to compliment the authors on their very clearly written and well-structured manuscript. The study is very relevant. I do have some concerns, mainly about the methodology and the intervention description and lack of behavioral analysis, as outlined below.

45 – Please be specific on the results in the abstract. In the conclusion, also be clear about the limitations of the study. How robust are your conclusions given the sample size?

86 – Can you provide more information about the context in Nepal? What are the mortality rates there, what issues are there with quality of care? What are the causes of this inadequate quality?

114 – What kind of algorithms are you referring to here?

166 – Perhaps you can add some information on previous research in other countries and about the expected contextual challenges of the clinics in rural Nepal. What are the behavioral mechanisms used?

122 – Unclear sentence, please rephrase.

140 – You are writing that the participants are pregnant women. In a way they are participants because their consent is needed to use their records for the study. However, from a behavioral perspective, it seems to me that the healthcare workers are the ones who are filling out the records, so they are the ones who are targeted with the intervention. Who were these HCW? How did they receive the intervention, do you have data on how many HCWs used the new system? Please be clear on the procedure and describe the EDSS.

146 – The sample size of 138 across 19 ANC centers and two different interventions concerns me. This bring me to my main methodological concern, which is that the data collection across different centers, and even across different healthcare workers, leads to levels in the data that should be analyzed as such. However, with only a limited number of cases per center, this is difficult. I'm interested in reading your view on the levels in the data and the potential effects on the outcomes, and potential alternative approaches that may tackle this.

301 – There is an interesting reflection on the behavioral aspect of the EDSS here, but I don't see this throughout the rest of the article. I would expect a rationale in the introduction or methods (description of the EDSS) where details the EDSS are explained, e.g. with regard to quality considerations, ease of use, implementation, etc. If this is described elsewhere, please refer to it in the article.

VERSION 1 - AUTHOR RESPONSE

Reviewer 1 comment	Authors' response	Page, line number in tracked changes version
First, I would like to compliment the authors on their very clearly written and well-structured manuscript. The study is very relevant. I do have some concerns, mainly about the methodology and the intervention description and lack of behavioral analysis, as outlined below.	Thank you for your positive feedback and for taking the time to review the manuscript.	
45 – Please be specific on the results in the abstract. In the conclusion, also be clear about the limitations of the study. How robust are your conclusions given the sample size?	Thank you for highlighting this important point. We have revised the results and conclusion section of abstract.	Page 2-3, line 50-56; page 3, line 59, 63-64
86 – Can you provide more information about the context in Nepal? What are the mortality rates there, what issues are there with quality of care? What are the causes of this inadequate quality?	Thank you for this point. We have added information about maternal mortality and quality of care in context of Nepal.	Page 4, line 86-87; line 92-97
114 – What kind of algorithms are you referring to here?	We acknowledge this phrasing and term was unclear. By algorithms, we were referring to the Nepal ANC guidelines-based	Page 5, line 120-121

	content of the EDSS to provide ANC consultation and manage high risk pregnancies. We have changed the phrasing in the manuscript.	
166 – Perhaps you can add some information on previous research in other countries and about the expected contextual challenges of the clinics in rural Nepal. What are the behavioral mechanisms used?	Thank you for your suggestion. To our knowledge, no studies have assessed the impact of EDSS on existing paper-based records. However, we have added a sentence in the Introduction regarding challenges facing EDSS implementation in similar low-resource contexts and how the EDSS was intended to work. We have also added an intervention section in the methods.	Page 5, line 125-130; page 6-7, line 162-176
122 – Unclear sentence, please rephrase.	Thank you. We have rephrased the sentence.	Page 5, line 136-144
140 – You are writing that the participants are pregnant women. In a way they are participants because their consent is needed to use their records for the study. However, from a behavioral perspective, it seems to me that the healthcare workers are the ones who are filling out the records, so they are the ones who are targeted with the intervention. Who were these HCW? How did they receive the intervention, do you have data on how many HCWs used the new system? Please be clear on the procedure and describe the EDSS.	Thank you for this important observation. We agree that healthcare providers were the participants in the mIRA project intervention and the targets of the desired behavioral changes. We have modified the manuscript to include a description of the intervention and to add references to other studies from the mIRA project evaluation. Several of these other papers report extensive details on how the intervention was delivered, include full descriptions of the EDSS and its theory of change, and report characteristics of the healthcare providers. We have tried to summarise key points about the EDSS intervention and the participating facilities and healthcare providers as concisely as possible in this manuscript.	Page 6-7, 162-176; page 7, 178

	However, we want to be clear to readers that the participants in this particular sub-study of the mIRA project were not the healthcare providers directly. Instead, this study analysed data extracted from pregnant women's ANC records. We hope the changes made to the manuscript address your important points raised.	
146 – The sample size of 138 across 19 ANC centers and two different interventions concerns me. This bring me to my main methodological concern, which is that the data collection across different centers, and even across different healthcare workers, leads to levels in the data that should be analyzed as such. However, with only a limited number of cases per center, this is difficult. I'm interested in reading your view on the levels in the data and the potential effects on the outcomes, and potential alternative approaches that may tackle this.	Thank you for your thoughtful comment. We acknowledge that data collection across different centers and healthcare providers and the implementation of two EDSS introduces potential levels in the data that the sample size is not fully able to account for. Unfortunately, the rural setting, where few pregnant women attend for ANC on any given day, severely restricted opportunities to increase the sample size of records (in each round) within the resource constraints of the project.<br><br>This is an important limitation of the study, and we have been cautious in our interpretation of the findings as a result. This study, being exploratory, focuses on understanding the overall impact of electronic records on existing paper-based records. We acknowledge that the study may be underpowered to detect smaller changes in completeness and agreement, but in developing the sample size targets we also considered what was a meaningful	Page 15, line 414-421 and line 435-436

	change in completeness/agreement in recordkeeping. We agree that the limited number of records per facility meant that we were unable to examine variation in change between participating facilities, including within EDSS allocations, and have modified the Discussion section to highlight this important consideration in future studies.	
301 – There is an interesting reflection on the behavioral aspect of the EDSS here, but I don't see this throughout the rest of the article. I would expect a rationale in the introduction or methods (description of the EDSS) where details the EDSS are explained, e.g. with regard to quality considerations, ease of use, implementation, etc. If this is described elsewhere, please refer to it in the article.	Thank you for this feedback. We have modified the manuscript to bring some of these points in throughout. We have added a new section to the methods on the intervention and also cited several papers where the behavioural mechanisms and details of the intervention implementation and effects are discussed in much greater detail.	Page 5, line 125-130; page 6-7, 162-174
